# Eating Disorders in the Era of the COVID-19 Pandemic: What Have We Learned?

**DOI:** 10.3390/ijerph182312381

**Published:** 2021-11-25

**Authors:** Palmiero Monteleone

**Affiliations:** Department of Medicine, Surgery and Dentistry “Scuola Medica Salernitana”, University of Salerno, 84081 Baronissi, Italy; pmonteleone@unisa.it; Tel.: +39-089672833

Eating disorders (EDs), including anorexia nervosa, bulimia nervosa, binge-eating disorder and other less frequent syndromes [1], are complex psychiatric conditions characterized by abnormal eating or weight-control behaviors and body image preoccupation [2], which affect mainly adolescent and young adult females [3]. Although their etiology is still unknown and likely involves biological, psychological and environmental factors [4], stressful experiences are widely acknowledged to represent putative risk factors for their development and maintenance [5]. Thus, the endogenous stress response system is thought to play a pivotal role in the pathophysiology of EDs [6], and evidence of an altered functioning of both components of the body stress response system, namely the hypothalamus–pituitary–adrenal axis and the sympathetic nervous system, has been reported in ED patients [7,8,9,10,11]. Furthermore, deranged cortisol, sympathetic and emotional responses to psychosocial stressors have been detected in ED patients [10,12], and this is consistent with the idea that an increased attentional bias toward social stimuli contributes to the pathophysiology of these disorders [13,14].

The outbreak of the coronavirus disease 2019 (COVID-19) pandemic with the consequent adoption of social and physical distancing measures to contain virus transmission has involved people’s exposure to different kinds of stressors, such as social isolation, impairment in the family and/or individual’s economic condition, disruption in routinary activities and in everyday life and fear of being infected [15,16]. Because of such a stressful situation, while some stress-related reactions, such as concentration difficulties, irritability, insomnia and interpersonal conflicts, have arisen as physiological responses to the pandemic [17,18,19], a clear-cut increased prevalence of pathological anxiety and depression has occurred in the general population [20,21,22]. In line with this, people with a pre-existing psychiatric disorder have shown heightened vulnerability in terms of physical and mental distress [23,24,25,26,27], while people with EDs have displayed heightened food restriction, an increase in excessive physical exercising and more frequent binge/purging episodes as well as a worsening of other ED-specific and internalizing symptoms (namely anxiety, depression and post-traumatic stress symptoms) [28,29,30,31,32]. These data are corroborated by the observed increase during the pandemic in the number of urgent and routine referrals of individuals with EDs [33] as well as by the increase in in-patient admissions, especially of adolescents with EDs [34,35,36].

Several factors have been associated with the worsening of psychopathology induced by the COVID-19 pandemic in ED individuals. Heightened isolation, fear of contagion, reduced satisfaction with family and friends’ relationships, reduced perceived social support and an increased exposure to aberrant routines and thin-related social media messages have been described as possible stress factors contributing to the deterioration of eating-specific and general psychopathological symptoms in people with EDs [31,32,37]. Moreover, changes in treatment delivery have also occurred during the COVID-19 pandemic since the imposed restriction measures have implied reduced access to in-person treatment and an increase in online treatments [38,39,40]. Even if in some cases online treatment generated the patient’s disappointment, since the therapeutic relationship was perceived as qualitatively unsatisfactory [38,39,40,41,42], in some others it allowed patients to maintain a therapeutic relationship [41] or to make treatment more accessible [41,42,43]. Thus, it has been demonstrated that the quality of the therapeutic relationship was inversely associated with the worsening of symptoms in ED people during the COVID-19 lockdown [32].

What can we learn from these COVID-19-related findings? First, the worsening of both ED-specific and general psychopathology during the stressful lockdown period supports the reliability of the hypothesized post-traumatic nature of ED symptomatology [10,12,44] and provides novel and consistent evidence for the existence of a transdiagnostic vulnerability to acute stressful experiences, since the pandemic-induced worsening of psychopathology has been replicated across different psychiatric populations [45,46,47]. Second, the pandemic-induced impairment of general psychopathological symptoms (i.e., disturbed sleep, anxiety, depressive, post-traumatic, obsessive compulsive and panic symptoms) together with the worsening of ED-specific symptomatology further supports the idea of a re-conceptualization of ED psychopathology as a broad spectrum of mutual relationships among non-specific and ED-specific psychopathology [48,49] and sustains the need for a personalized approach in the treatment of people with EDs as it is acknowledged for other psychiatric conditions [50]. Third, the data on COVID-19-induced changes in treatment delivery and the inverse association of patients’ perception of the quality of telehealth medicine with the worsening of symptoms confirm the role of the therapeutic alliance as one of the most important resilience factors for individuals with EDs [51]. Fourth, the widespread implementation of internet-delivered psychological treatments induced by the COVID-19 pandemic [52,53], associated with the relatively positive attitudes toward e-therapies of people with EDs [54], corroborates the idea that, at least in some cases, telehealth therapies could be a promising opportunity to treat severe psychiatric disorders.
